# Exploring the effects of adiponectin and leptin in correlating obesity with cognitive decline: a systematic review

**DOI:** 10.1097/MS9.0000000000000766

**Published:** 2023-05-04

**Authors:** Nishat Tasnim, Nawsheen Khan, Aditi Gupta, Purushottam Neupane, Aashna Mehta, Shahtaj A. Shah, Rohit C. Dey

**Affiliations:** a North Bengal Medical College, Sirajgonj, Rajshahi, Bangladesh; b Caribbean Medical University, Curaçao, The Netherlands; c Jawaharlal Nehru Medical College, Belgaum, Karnataka, India; d Punjab Medical College, Faisalabad, Punjab; e Jinnah Medical and Dental College, Karachi, Pakistan; f Faculty of Medicine, University of Debrecen, Debrecen, Hungary; g Altai State Medical University, Altai Krai, Barnaul, Russia

**Keywords:** adiponectin, Alzheimer’s disease, brain awareness, cognitive decline, dementia, leptin, lifestyle modification, memory loss, obesity, obesity prevention

## Abstract

Obesity and cognitive decline including dementia and Alzheimer’s Disease (AD) affect millions worldwide. Several studies have shown that obese individuals suffer from cognitive decline. Here, we suggest that adiponectin and leptin, protein hormones secreted by white adipose tissue explain the relationship between obesity and cognitive decline. We systematically searched PubMed and World Health Organization (WHO) websites with the keywords obesity and dementia and compiled literature that explains how adiponectin and leptin impact obesity and cognitive decline. Full-text, free-access articles on PubMed published after 2009 have been included. Whereas articles published before 2009, books, and reports were excluded. We concentrated on mechanisms via which adiponectin and leptin affect energy expenditure, fatty acid catabolism, satiety, hunger, Body Mass Index (BMI), neurogenesis, and brain structures that lead to the development of cognitive dysfunction. Moreover, we hypothesized that adiponectin and leptin hormones explain how obesity and dementia are connected. After compiling the research studies, we summarized that adiponectin and leptin negatively correlate to BMI. Adiponectin arbitrates energy expenditure and fatty acid catabolism to prevent obesity. In the presence of adiponectin, hippocampal cells proliferate, whereas neurogenesis is reduced in its absence. However, leptin prevents obesity by promoting satiety, reducing hunger, and increasing insulin sensitivity. It also has neuroprotective effects thus reducing the risk of developing cognitive decline. So, physical exercise, diet alteration, weight reduction, adiponectin, and leptin supplementation should be carried out to protect against obesity-induced cognitive decline. Therefore, further research studies should be done in this area.

## Introduction

Highlights

- People with obesity demonstrate higher cognitive dysfunction.
- Adiponectin and leptin are the two major hormones that explain how obesity and cognitive decline are interconnected.
- Adiponectin prevents obesity by mediating energy expenditure and fatty acid catabolism. While it protects against cognitive decline by promoting neurogenesis, mediating synaptic function, and reducing oxidative stress.
- Leptin prevents obesity by reducing hunger, promoting satiety, increasing insulin sensitivity and lipolysis, whereas, it protects against cognitive decline by preventing neurodegeneration and obesity-induced leptin resistance.
- Combined physical exercise, diet and weight alteration, and adiponectin and leptin supplementation, along with cognitive stimulations, help in the prevention of cognitive deficits.

According to recent data, 38.5% of the U.S. population is obese^1^. 2.8 million adults die each year due to conditions associated with obesity, according to data published by the World Health Organization (WHO)^2^. Dementia, which is the seventh leading cause of death among all other morbidities, affects 55 million people worldwide^3^. The first-ever research to have shown an association between obesity and dementia was conducted in 2003. The study concluded that obesity is a leading cause of dementia, more commonly Alzheimer’s disease (AD)^4,5^. To explain how dementia and obesity are connected, we focus on the role of leptin and adiponectin. Both leptin and adiponectin are protein hormones secreted by adipose tissue that have a protective role against obesity and dementia^6^. Adiponectin is a key player in fat catabolism and has an important role in energy expenditure, hence it leads to weight loss^7^. Therefore, we can conclude that adiponectin prevents obesity. A study conducted on rodents has shown that high-fat-fed mice were seen to have lower levels of adiponectin compared to normal fat-fed mice. Whereas, lower levels of adiponectin lead to more cognitive decline in the high-fat-fed mice. Hence, it can be concluded that dysregulated adiponectin is responsible for developing obesity and cognitive decline. To explain further how the decreased level of adiponectin causes cognitive decline, the paper focuses on the role of two important receptors in the hypothalamus, AdipoR1 and AdipoR2. Once adiponectin binds to these receptors, it protects neurons from oxidative stress. Lack of adiponectin means decreased protection for the neurons. It results in spatial memory and learning deficits which are seen in cognitive decline^6^. Secreted by adipose tissue, leptin prevents obesity and has a neuroprotective function as well. Leptin promotes satiety and reduces hunger. Thus, it prevents obesity. Besides, low-grade inflammation in obesity leads to leptin resistance. Leptin resistance further develops disruptions in the signaling pathways and obesity. The absence of leptin also leads to neurodegeneration, as leptin plays an important function in neuron configuration. Higher the neurodegeneration, the higher the chance of developing cognitive decline. Moreover, this paper discusses the detailed mechanisms by which leptin links the development of obesity and dementia^8^.

The purpose of this systematic review is to analyze the effects of adiponectin and leptin on the brain and how they correlate to the development of cognitive decline in obesity. We hypothesize that both adiponectin and leptin play significant roles in associating these two diseases. Moreover, this study aims to conduct a critical appraisal of existing literature that shows the connection between obesity and dementia via leptin and adiponectin. Until now, most studies have shown how obesity and other conditions such as Type 2 Diabetes Mellitus (T2 DM), insulin resistance, heart failure, and Metabolic Syndrome (MetS) are linked to dementia. Also how the dysregulated adiponectin and leptin are developing cognitive decline. Our study is unique because it focuses solely on dementia and obesity, in its attempt to explain why there is a high prevalence of dementia in the obese population via adiponectin and leptin. This review goes into detailed mechanisms of how low-grade inflammation in obesity leads to neurodegeneration. It also emphasizes different signaling pathways, and how brain structures are affected by adipokines. While most literature discusses the effects of not just leptin and adiponectin but also TNF-alpha, interleukin-6, plasminogen activator inhibitor-1, and angiotensinogen, this paper only highlights the roles of adiponectin and leptin in detail. However, very little literature is available on the epidemiologic association of dementia and obesity with a focus on adiponectin and leptin. We believe this review is a compilation of important findings on the subject matter. Furthermore, we only reviewed articles that have free access, hence not all literature on this topic has been screened. Apart from diet and physical activity, the literature did not take into consideration the impacts of age, ethnicity, gender, lifestyle, etc., in explaining the association between obesity and dementia. So, future studies should be done taking these factors into consideration.

## Methodology

**Figure 1:**
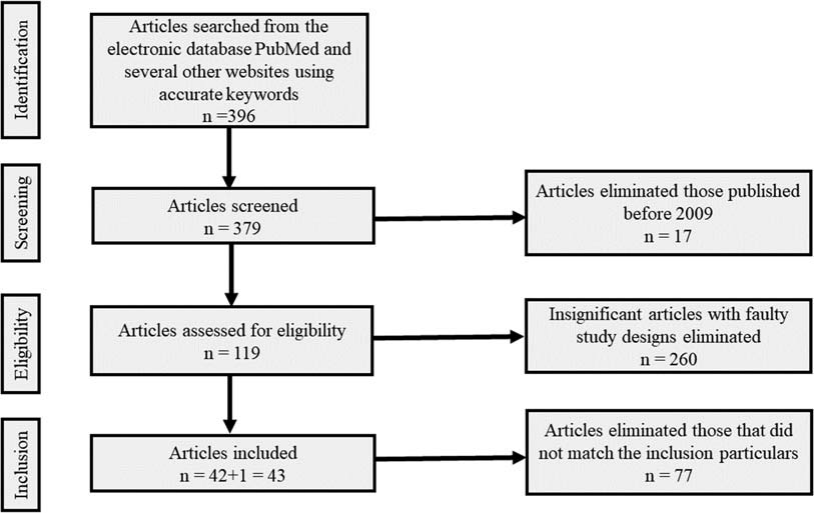
PRISMA (Preferred Reporting Items for Systemic Reviews and Meta-Analysis) protocol showing screening procedure^9^.

This review was conducted according to the PRISMA (Preferred Reporting Items for Systemic Reviews and Meta-Analysis) guidelines and AMSTAR (Assessing the methodological quality of systematic review) guidelines^9^. PRISMA guidelines also helped us to find the appropriate studies for this article, which is displayed in Figure 1. Moreover, this paper has been registered with the National Institute of Health Research, and the registration’s unique identifying number is CRD42022371083.

### Criteria for study selection

#### Types of studies

Experimental, randomized controlled trials (RCT), non-randomized studies of interventions (NRSI), reviews, systematic reviews, and meta-analyses conducted on humans and animals.

#### Types of participants

Humans and animals.

#### Types of outcome measures

Primary goal of the article was to determine how adiponectin and leptin play a role in connecting obesity with dementia.

### Search methods

#### Selection of studies

During our review, we searched PubMed and some other databases with the keywords ‘Obesity’ and ‘Dementia’. This yielded a total of 398 articles (396 from PubMed and two from WHO), which were carefully reviewed and cross-examined by the authors for this research.

#### Inclusion and exclusion criteria

Inclusion criteria established for the manuscript included articles from the last 13 years (2022–2009) and full-text English-language articles with free access to PubMed. We searched the database with the keywords ‘Obesity’ and ‘Dementia’.

Studies that did not meet these criteria were excluded.

#### Data collection

Two reviewers worked independently to scrutinize the eligibility of each paper and record. Disputes were resolved by discussion. After extensive screening and cross-examination, 42 research papers were selected. However, with another one for PRISMA and AMSTAR guidelines, a total of 43 studies were incorporated into our paper. Important findings from 16 articles were tabulated in the Results section. Distinct varieties of studies have been incorporated to add additional outcomes. Certain kinds of literature such as books, journals, letters to editors, case reports as well as articles with faulty designs, those that did not have relevant information to answer our research question, and articles published before 2009 were eliminated. To avoid bias and eliminate any confounders, only articles with the mentioned keywords were reviewed to find the association between obesity and dementia.

### Data extraction, analysis, and synthesis

All the authors were involved in the data analysis, and each of them extracted data from each paper in the preliminary stage. Then two authors reviewed all the extracted data and added significant findings if any were missed. A wide variety of outcomes were found. We mostly focused on

1. How obesity is connected to cognitive decline.
2. How different adipokines such as adiponectin and leptin affect obesity and cognitive decline.
3. What measures can be addressed to prevent obesity and cognitive decline.

We pointed to the particular outcomes of studies that matched the specific sections. Studies with similar outcomes have been tabulated together according to their sequence of description in the paper.

## Results

Among a large number of studies, we have thoroughly revised and added a total of 43 articles. Some other studies met the inclusion criteria; however, due to faulty study design and poor outcomes, those have been excluded. Crucial findings from 16 articles have been added in Table 1.

**Table 1:** Crucial findings of literature review.

Author and year of publication	Study design	Summary	Outcome	Limitation
Espeland *et al.*, 2018^10^	RCT	An RCT was conducted on 1091 random people. The experimental group (obese people) was induced to extensive modifications of lifestyle. Whereas, the control group (normal weight) was given diabetes treatment and education, as they had T2 DM. Both groups were assessed 10 years later.	Obese people manifested more cognitive decline than normal people.	N/A
Dye *et al*., 2017^11^	Review article	The correlation between obesity and cognitive function was assessed. The study emphasized both the immediate effects of cognitive function and the long-term effects of cognitive aging and the subsequent likelihood of the development of dementia. The review not only assessed this relationship but also identified stress as an important hazard for the advancement of abdominal adiposity; this in turn contributed to a reduction in cognitive performance.	The presence of obesity with its comorbidities did show an increase in the risk of a decline in cognitive function and the development of dementia.	Females had higher body fat compared to men having the same BMI. Also, the difficulties in differentiating muscle from fat tissue when calculating BMI limited the assessment of true body fat. Moreover, The length of the follow-up period makes drawing conclusions difficult when this is limited.
Alosco *et al*., 2014^12^	Review article	The purpose of this study is to evaluate adiposity as an important risk component for adverse brain changes and cognitive deterioration in patients with heart failure.	Obesity is a potential contributor to cognitive damage in heart failure patients.	Generalizability is limited due to the specific study methodology.
Stanek *et al.*, 2013^13^	Review	A review focused to assess the risk reduction of AD after bariatric surgery in obese patients. Obesity may result in structural brain modifications that bring the furthered risk of dementia, cognitive impairment, and poor neurocognitive outcomes. Bariatric surgery can be a beneficial management option for obesity. Thus, it improves neurocognitive function.	Obesity is directly related to declining in specific cognitive function and an increased risk of dementia. Bariatric surgery may assist to relieve some of the cognitive dysfunction in adults. Thus, it seems to be an attainable and favorable option for lessening obesity-associated cognitive damage.	There is a lack of longitudinal cognitive evaluation and there are also reported variations in perioperative complications.
Kim *et al.*, 2017^14^	Experimental study	An experimental study was done on AdipoR1 and AdipoR2 knockout mice to explore the effects of adiponectin on AD-like disease. They used gene therapy-guided shRNA suppression for this experiment.	AdipoR1 suppression promotes neurodegeneration. AdipoR1 knockdown mice demonstrated higher body weight. Also, adiponectin supplementation profoundly decreases brain degeneration.	N/A
Kiliaan *et al.*, 2014^15^	Review article	A review assessed the relationship between what factors of obesity (higher BMI) contribute to the risk of development of dementia, and overall brain health.	A possible correlation between obesity and dementia includes the potential increase in adipose tissue hormones and adipokines due to the increased adipose tissue in obesity. Two times increase in the risk of dementia was seen in midlife or centrally obese people.	The complexity of adipokines’ function and their nature of being secreted from not only adipose tissue yields them as poor indicators of neurodegeneration in relation to adipose tissue.
Rizzo *et al.*, 2020^16^	Review article	This review was conducted to assess the current development in the treatment strategies of AD. It attempts to analyze the use of adiponectin as a therapeutic intervention for obesity and AD. Further, it focuses on the various neuroprotective functions of adiponectin. It also discusses that an effective method for the reduction of the risk of AD is through the avoidance and treatment of obesity.	This study endorses that a therapeutic intervention for AD could be adiponectin.	N/A
Mendiola-Precoma *et al.*, 2016^17^	Review article	This review discusses the risk factors and pathogenesis of AD. It also provides a description of pharmacological and non-pharmacological treatment options (lifestyle, etc.).	Obesity is an important risk factor for AD. It involves adipokine dysregulation, which results in the release of the pro-inflammatory adipokines and decreases anti-inflammatory adipokines. Therefore, lifestyle modifications such as exercise can help to prevent AD.	
Lee and Mattson, 2014^18^	Review article	This article determines whether obesity can be linked to detrimental changes in the brain. It also discusses the properties of adipokines that lead to the pathology of the brain. It tries to relate the disease to the capacity of adipokines that enter the blood–brain barrier as opposed to adipokines that cannot. It further discusses the effect of adiponectin and leptin on dementia.	This study suggests that brain pathology can be linked to obesity. A number of adipokine actions on the brain increases the emergence of pathological processes in the brain.	N/A
Bednarska-Makaruk *et al.*, 2017^19^	Investigational study	This study included participants with different types of dementia, and they were compared with controls with normal cognitive function. Adiponectin, leptin, resistin, pro-inflammatory markers, vitamin D, cholesterol, and markers of glucose metabolism were assessed.	Dementia due to neurodegenerative disease is associated with adiponectin, leptin, and resistin. Identification of the role of resistin as a biomarker may help in the prevention of dementia.	Age and level of education, and physical activity needs to be controlled. The sample size for leptin analysis is small, whereas isoforms are not studied.
Flores-Cordero *et al.*, 2022^8^	Review article	Leptin, an adipokine increase in obesity, has a role in the hypothalamus, CNS nuclei such as the cerebral cortex and hippocampus. These regions are the first to be affected by chronic neurocognitive deficits containing the long-acting leptin receptor, a unique receptor capable of completing leptin signaling. Leptin resistance due to obesity is an important risk factor for AD. Obesity is a chronic low-grade inflammatory state responsible for leptin resistance through various mediators.	Leptin, through its action in the periphery and CNS, produces neuroprotective effects that improve cognitive function and memory. Its dysfunction in signaling pathways and resistance can cause neurodegeneration. Obesity-related inflammation may contribute to leptin resistance. Anti-inflammatory drugs combined with drugs that reduce leptin resistance could be useful for the treatment of AD.	N/A
McGuire and Ishii, 2016^20^	Review article	This review determines the relationship between leptin dysfunction and the development of AD due to its influence on hypothalamic neural circuits.	Normal leptin function protects against neurocognitive decline. Leptin dysfunction can aid in neurocognitive decline and AD. However, worsening AD can also interfere with leptin signaling.	N/A
Szabo-reed *et al.*, 2019^21^	RCT	An RCT was conducted to analyze the impact of pharmacological and non-pharmacological interventions for the prevention of dementia. Diabetes adversely affects cognitive function. Weight loss through increased physical activity and by reducing caloric intake can improve cognition.	The combined effects of exercise and intensive pharmacological and vascular risk factors for 2 years provide greater benefits as compared to either alone.	N/A
Scarmeas *et al*., 2009^22^	Investigational study	An investigational study was carried out to find out the interrelated association of diet and physical activity with the risks of AD.	A Mediterranean-type diet along with intense physical activity could reduce the risk of AD.	N/A
Espeland *et al*., 2018^23^	RCT	An RCT was conducted on the basis of the potential preservation of higher cognitive function in 5084 overweight individuals with T2 DM by intensive lifestyle modifications. The lifestyle modifications are aimed at both reduction of weight and increased activity.	Long-term intensive lifestyle modifications may have the potential to prevent higher cognitive function decline in adults with T2 DM. However, no prevention was found in those who experienced cognitive decline prior to the study.	N/A
Smith *et al.*, 2010^24^	RCT	Participants with high BP and obesity were randomized to DASH (Dietary Approaches to Stop Hypertension) diet alone, DASH+exercise+calorie restriction. Usual diet measured the executive function memory and psychomotor speed at the beginning and end of 4 months.	DASH+exercise+calorie restriction showed the best improvement in neurocognitive function.	Smaller sample size. Long-term outcomes were not measured. Also, no control group was present.

AD, Alzheimer’s disease; BMI, body mass index; CNS, central nervous system; N/A, not applicable; RCT, randomized controlled trial; T2 DM, Type 2 diabetes mellitus.

## Discussion

During the literature review, we found that people with obesity demonstrated higher cognitive dysfunction than those with normal BMIs^10^. Moreover, patients with other comorbidities such as heart failure and T2 DM possess a significantly greater risk of developing cognitive deficits^11–13^. Studies suggested that adiponectin could correlate with obesity and dementia as AdipoR1 and AdipoR2 suppression promotes neurodegeneration^14,15^. Also, twice the increased risk of dementia was seen in those with central obesity^15^. Moreover, AdipoR1 knockdown mice demonstrated higher body weight^14^. Researchers also demonstrated that adipokine dysregulation promotes obesity and the progression of AD^16–19^. Besides, adiponectin supplementation profoundly decreases neuronal degeneration and restores impaired hippocampal neuron proliferation. So, it could be used as a therapeutic intervention^14,16,17^. Furthermore, Flores-Cordero *et al*.^8^ showed that obesity promotes leptin resistance. Leptin also acts in the central nervous system (CNS) and produces neuroprotective effects that improve cognitive function and memory^8,20^. So, lifestyle modifications such as diet change, exercise, and therapeutic interventions such as adiponectin and leptin supplementation may protect against obesity and, thus, obesity-induced memory loss^16,21–24^.

Obesity, or excessive body weight, is a pathologic condition and a global epidemic^18^. In recent years, the prevalence of obesity has been on a continuous rise globally^25^. This can be linked to unhealthy eating practices and a diet containing a high glycemic index and a high fat content^25^. It is attributed to a sedentary routine and overnutrition^18^. Also, diet and exercise can have a direct influence on modifying the chances of becoming obese^26^. A vast range of chronic illnesses has been directly related to being obese^27^. Contrary to BMI, indices of central adiposities, such as waist size, have been proposed to be more useful indicators of the risk linked to obesity^27^. Obese persons have been reported to have greater chronic low-grade inflammation in adipose and several other tissues, as well^25^. Currently, there are 20 million dementia sufferers globally, and predictions indicate that the number will increase twice the figures by 2030^28^. The most frequent kind of dementia, accounting for over 60% of cases, is AD. These estimates are worrisome since 40% of occurrences may be avoidable and may have preventable causes^26^. AD can be linked to memory loss, cognitive and speech deficits. It is also related to the diminishing of other cognitive areas and difficulty in carrying out everyday activities^28^. Other main types of dementia include Lewy Body disease, vascular brain injury, and frontotemporal dementia. There are, however, instances where more than one type of dementia has co-occurred as well^25^. A meta-analysis conducted in China and USA suggested that dementia is a common complication seen in obese individuals. It suggested that an increasing number of obese persons are likely to be associated with a rising number of dementia cases in the coming years^28^. Rizzo *et al*.^16^ showed that obesity has a detrimental effect on the glial neuronal crosswalk and gives rise to obesity. It has been proposed that an inflammatory mechanism connects obesity to neurological diseases^25^. According to evidence, neurodegeneration can be exacerbated by peripheral inflammation^26^. Furthermore, obesity has been linked to an enhanced chance of developing cognitive decline according to a meta-analysis conducted on 2.8 million individuals^27^. Each dementia subgroup has a specific link to body size and weight fluctuation. According to Lee *et al*.^27^, there is a need to advocate for weight management and minimize weight fluctuations throughout adulthood.

White adipose tissue (WAT) is a compound tissue composed of distinct varieties and phenotypes of cells that depends on the structure and location of those cells^15^. The word adipokine (‘adipo- adipose, cyto- cell, and kinos- movement’) refers to the immunomodulatory cytokines produced by adipose tissue^15^. Adipokine consists of polypeptide hormone, which is a part of the complement 1q family^16,28,29^. Adipose secretome or adipokine, a distinct type of WAT cell secretes this hormone^30^. Visceral WAP releases more adipokines compared to the subcutaneous tissue. So, it can be used as a tool for measuring body adiposity, as it is related to waist circumference^15^. Furthermore, various kinds of adipokines take part in different pathways. Adiponectin, leptin, and adipsin mainly perform energy expenditure, metabolism, and fatty acid catabolism. While interleukins and monocyte chemotactic proteins help in the inflammatory response, plasminogen activator inhibitor-1 modulates thrombosis and hypertension^15,16^.

## Adiponectin

Adiponectin is a complex multimeric protein composed of high molecular weight hexamers and trimers. The effects of adiponectin are modulated by two receptors, AdipoR1 and AdipoR2^7,15^. These are expressed in the adipose tissue, hippocampus, hypothalamus, pituitary gland, brainstem, and so on^3,15^. Major signaling molecules activated by adiponectin are 5′ adenosine monophosphate-activated protein kinase (AMPK), peroxisome proliferator-activated receptor-α (PPARα), IkB kinase (IKK)/NF-κB/PTEN, IRS1/2–Akt, and Ras-ERK1/2, p38MAPK, and ERK1/2-MAPK^7^. Adiponectin mediates energy expenditure, food intake, inflammatory process, glucose homeostasis, and fatty acid catabolism^15,29^. In the hypothalamus, it mediates food consumption and energy expenditure via AdipoR^16^. It also takes part in fatty acid catabolism in the liver and skeletal muscle by activating acetyl coenzyme-A carboxylase (ACC) and AMPK^16^. With the reduction of plasma adiponectin level, this entire regulation is hampered. Eventually, WAT and surrogate measures such as body fat percentage increase, which gives rise to obesity^7^. In favor of this, Kim *et al*.^14^ has proposed through their rodent study that adiponectin counteracts weight gain by activating energy expenditure. Moreover, Kim *et al*.^7^ suggested that, with the elevation of central adiposity, adiponectin level reduces. This mechanism further worsens obesity. That means adiponectin level is negatively correlated with obesity^7,14,19^. So, it can be said that adiponectin prevents obesity by reducing food intake, mediating energy expenditure and fatty acid catabolism as shown in Figure 3 ^15,16^. Furthermore, adiposopathy, defined as excess adipose tissue hypertrophy, happens to be seen in obese and elderly people^31^. It disrupts adiponectin regulation and inhibits paracrine and endocrine adipose tissue function^31^. This dysregulation of adipose tissue gives rise to frailty syndromes including cognitive dysfunction, sarcopenia, and decreased daily activity^32^. However, Ishii and Iadecola^33^ demonstrated that the effects of adiponectin in relating age to dementia are not well established. Whereas, Arnoldussen *et al*., Kiliaan *et al*., and Ng and Chan^6,15,34^ demonstrated a significant association of adiponectin in relating obesity to dementia.

**Figure 2:**
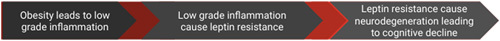
Effects of leptin^8^.

**Figure 3:**
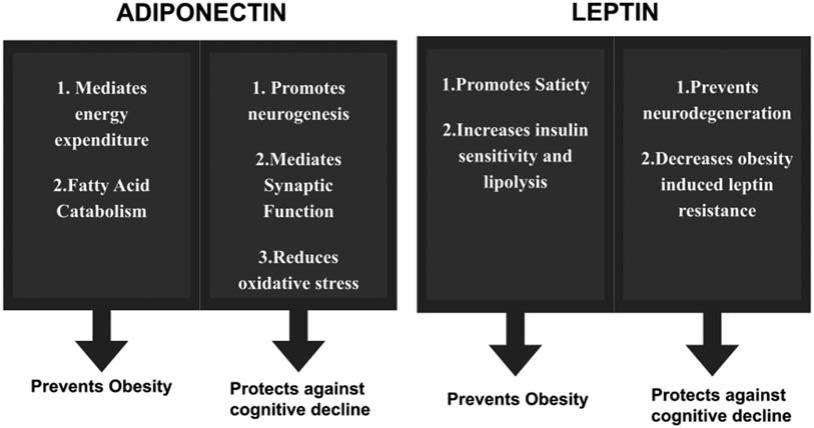
Showing the effects of adiponectin and leptin in correlating obesity and cognitive decline^7,8,15,16,29,37^.

Moreover, AdipoR1 and AdipoR2 are two varieties of adiponectin found in both the hypothalamus and the basal nucleus of Meynert^7^. Adiponectin assists in neurogenesis through AdipoR1 and mediates synaptic function through AdipoR2. It works in the energy balance pathway and higher brain function. It also protects neurons against oxidative stress^16,34^. In addition, spatial memory and learning deficits happened to be seen in mice with adiponectin insufficiency^34^. Chan *et al.* showed that adiponectin had preventive effects against cytotoxicity caused by oxidative stress on human cells afflicted with Amyloid-β (Aβ) neurotoxicity^16,35^. Whereas, Rizzo *et al*.^16^ demonstrated that hippocampal progenitor cells in adults proliferate in the presence of adiponectin by activating p4 MAPK, while neurogenesis is reduced in the absence of adiponectin. Another recent clinical study has exhibited better cognitive function in people with above-average adiponectin levels. Besides, the mice study also displayed greater brain infarctions and higher neurologic deficits after ischemia in adiponectin knockout mice in comparison to the wild type. In addition, Adiporon, an adiponectin agonist, binds to the AdipoRs receptor. Studies demonstrate that adiporon improves cognitive dysfunction, restores impaired hippocampal neuron proliferation, and inhibits Aβ deposition by activating the AdipoR1/AMPK pathway^7^. It has also shown evidence of reduced brain degeneration and infarction size^14,16^. So, it may be used as a therapeutic intervention for obese people^7,14,16^. Thus, it can be said that adiponectin protects against cognitive decline by promoting neurogenesis, mediating synaptic function, and reducing oxidative stress as shown in Figure 2 ^16^. That means adiponectin is related to both obesity and cognitive decline via AdipoR1 and AdipoR2 receptors. As adiponectin dysfunction gives rise to obesity, it can also be said that obesity can cause cognitive decline too. In light of this, one study demonstrated that high-fat diet-fed mice accelerated more cognitive deficits in comparison to normal diet-fed mice^7^. Whereas, another study revealed that a high-fat diet for a longer time in the knockout mice manifested decreased amount of adiponectin leading to cognitive dysfunction^29^. Thus, it can be concluded that obesity and cognitive decline are interrelated via adiponectin as shown in Figure 3 ^7,16,29^.

## Leptin

Leptin, one of the adipokines, is a hormone predominately made by adipose tissue and enterocytes in the intestine. It balances the intake of food and energy expenditure, enhances insulin sensitivity, increases lipid breakdown, and inhibits lipid synthesis which is negatively correlated with BMI. Leptin and insulin both have receptors on proopiomelanocortin (POMC) neurons in the hypothalamus that help to manipulate energy balance and glucose homeostasis^15^. Peripheral leptin crosses the blood–brain barrier (BBB) and choroid plexus. It then enters into the brain and cerebrospinal fluid and acts on various areas of the brain, particularly on the hypothalamus and hippocampus. Some leptin can also be generated in the human brain^16^. Once in the brain, leptin regulates the functions of pancreatic beta cells by promoting insulin sensitivity, provoking beta cell proliferation, and lowering beta cell apoptosis. Studies also suggest that leptin along with other adipokines directly acts on hypothalamic nuclei such as arcuate nuclei^15^. Furthermore, leptin produces orexigenic neuropeptide Y (NPY), anorexigenic melanocyte stimulating hormone (MSH) along with Agouti-related peptide (AGRP) that regulates energy expenditure and food intake. It also stimulates satiety in the medial hypothalamus by promoting MSH synthesis. In the lateral hypothalamus, it inhibits hunger by producing orexigenic NPY. Moreover, metreleptin, a leptin analog, increases insulin sensitivity by at least five to seven-fold^36^. It has also been shown to reduce the activation of the hunger centers such as the insula, parietal and temporal cortex, and improves the activation of the satiety center prefrontal cortex^36.^ Hence, by promoting satiety, reducing hunger, increasing insulin sensitivity, and mediating lipolysis, leptin plays a protective role against obesity as shown in Figures 2 and 3 ^36^.

Leptin is associated with the structure, neuronal configuration, and function of the brain^15,20^. Learning and memory processes are partly mediated by leptin in the various hypothalamic and hippocampal regions^15^. It also plays an important role in the neuroendocrine-immune function^15^. However, obesity causes systemic inflammation, which leads to leptin resistance. However, leptin resistance is one of the major factors in neurodegeneration. As neurodegeneration worsens, it leads to the development of cognitive decline^8^. In favor of this, Flores-Cordero *et al*.^8^ exhibited that neurodegeneration is caused by leptin resistance. To understand how obesity leads to leptin resistance and how leptin resistance causes AD to progress, we shed light on two mechanisms: inflammation and signaling pathway. Low-grade inflammation persistent in obesity increases the amount of human C-reactive protein (CRP). CRP decreases the amount of leptin that enters the CNS, subsequently altering the physiologic function of leptin leading to leptin resistance. Inflammation also leads to the increased expression of the intracellular factor SOSC3. Increased SOSC3 inhibits the JAK2/STAT3 pathway, which in turn causes leptin resistance^8^. Besides the hypothalamus, other areas of the brain such as the cerebral cortex, hippocampus, and dentate gyrus contain the long-term leptin receptor, LepRB. These are also the earliest regions affected by neurocognitive disorders such as dementia. LepRB signals via the STAT3 pathway. However, genes such as SOCS3, PT1B, and TCPTP act through the mechanism of Tyr98 phosphorylation on LepRB. The cytokine suppressor signaling 3 also plays a major role in leptin resistance^8^.

Moreover, the amount of gray matter in various areas of the brain is directly linked to plasma leptin level. The defect in leptin signaling leads to the worsening of cognitive ability and favors the pathology of the development of dementia^20^. An experimental study by Chacrabarti *et al.* on transgenic mice concluded that leptin may influence the amyloid precursor protein A (APP-A). Beta regulation deficiency of leptin causes A beta accumulation, beta-secretase expression, and phosphorylation of tau proteins, which cause neuronal death leading to the development of AD^7,8^. Moreover, leptin analogs such as metreleptin and myalept have shown efficacy in treating people with very low levels of leptin. Patients suffering from congenital leptin deficiency have benefitted from leptin analog treatment as it improves their obesity and cognitive deficiency^36^. So, it can be concluded that leptin prevents obesity. It also prevents cognitive decline by protecting against neurodegeneration and obesity-induced leptin resistance as shown in Figures. 2 and 3 ^7,8,37^.

Considering all these, researchers are working tirelessly to find better preventive modalities that might reduce the risk of obesity and cognitive impairment. Smith *et al*.^24^ showed that a combined DASH (Dietary Approaches to Stop Hypertension) diet, calorie restriction, and aerobic exercise improve neurocognitive function among sedentary overweight individuals. However, Amanda *et al.* showed that physical exercise, cholesterol reduction, and blood pressure control enhance global cognitive function by slowing brain atrophy and improving structural and functional connectivity of the brain^2^. Besides, several other studies also manifested improvement in higher cognitive functions in physically active patients or those consuming a rye-based or Mediterranean diet^22,23,38,39^. Moreover, Kivimaki *et al.* demonstrated that elderly people who were doing cognitively stimulating jobs manifested lower onset of dementia in comparison to those who were doing non-cognitively stimulating jobs. The reason could be that cognitive stimulation reduces the level of plasma proteins, which lowers axonogenesis and synaptogenesis as well as elevates the risk of dementia^40^. Also maintaining social engagement might decrease the risk of dementia in the elderly^34^. Moreover, adiponectin agonist adiporon improves cognitive dysfunction, restores impaired hippocampal neuron proliferation, and inhibits Aβ deposition, by activating the AdipoR1/AMPK pathway^7^. So, it may be used as a therapeutic intervention for obesity^7,14,16^. Leptin analog metreleptin can also be a good option for obese people. As it reduces the activation of the hunger centers located in the insula, parietal and temporal cortex and improves the activation of the satiety center in the prefrontal cortex. Thus, it helps to reduce obesity^36^. Furthermore, studies also suggested that bariatric surgery has been proven to induce quick gains in memory and executive function and lasts for years after surgery^13^. However, there are some detrimental effects of these interventions. The DASH diet may cause bloating. Studies have shown more bloating in men than in women^41^. Rye-based diet has been shown to elevate low-density lipoprotein (LDL) and triglyceride levels^42^. Moreover, chronic adiponectin upregulation may cause heart damage due to left ventricular hypertrophy. It has also been shown to promote angiogenesis leading to tumor growth. Infertility can also be triggered by chronically increased adiponectin levels^43^. According to Paz-Filho *et al.*, common side effects that were reported with leptin supplements included headache, hypoglycemia, decreased weight, and abdominal pain. Moreover, people with acquired generalized lipodystrophy taking leptin supplements reported the development of T-cell lymphoma. However, a causal relationship between the supplement and T-cell lymphoma has not been reported. Previous studies revealed lymphomas have been seen in both familial partial lipodystrophy and acquired generalized lipodystrophy, irrespective of taking the supplements^36^. Bariatric surgery can develop major nutritional deficiencies, bone demineralization, recurrent infections, gallstone formation, hernia, and premature birth in pregnant women^13,24^. Furthermore, calorie restriction, diet modification, physical and aerobic exercises, cholesterol reduction, and BP control seem very practical and feasible for the normal population. These interventions are generally needed to keep a healthy lifestyle. However, adiponectin, leptin supplementation, a rye-based diet, and bariatric surgery should be considered according to their risk benefits ratio.

In short, obesity accelerates neurocognitive dysfunction^1,28^. However, adiponectin and leptin play a protective role against obesity and thus prevent obesity-related cognitive decline^15–20,32–34^. Hence, combined physical aerobic exercise, diet and weight modification, and adiponectin and leptin supplementation, along with cognitive stimulations, might aid in the prevention of cognitive deficits^16,21–24,38–40^.

The main strength of our article is that it was conducted on a very narrow topic with specific goals, that is to find the link between obesity and dementia via adiponectin and leptin. Most studies published so far have not focused on these two diseases only but on other diseases such as T2 DM, MetS, and insulin resistance as well. The study only included information that met the inclusion and exclusion criteria, which adds to the strength of our review. Keywords were carefully decided in the beginning, and the search was performed based on the keywords. Moreover, this study takes into account a large sample size, which strengthens the outcomes. All these articles were carefully screened and cross-checked by applying the PRISMA guidelines to discover and filter out the relevant 43 articles. Critical findings from 16 articles are shown in Table 1, where the main findings, summary, and limitations of the studies have been summarized.

Considering limitations, during the review, only one database was utilized, which limited the amount of literature available. In addition, only the articles with free full text were taken into consideration. The keywords were limited to ‘obesity’ and ‘dementia’. Eventually, only 43 articles were selected for data extraction. Although clearly defined by selection criteria, these factors possibly limited the amount of data available to make comprehensive evaluations. Apart from diet and physical activity, demographic characteristics such as age, gender, race, and ethnicity did not influence obesity-related dementia.

In this part of the discussion, we aim to compare our work with the existing literature and also among the existing literature along with their strengths and limitations. Out of the 43 studies we reviewed, small sample size is a predominant limiting factor^19,24^. Small samples with mismatched ages, education levels, and lack of generalizability lead to the development of bias^19,24^. Dye *et al.* demonstrated that the association between being underweight in midlife and the risk of developing cognitive decline in later life is prone to ascertainment and selection bias. They also mentioned that the use of self-reported data for height and weight at an earlier age may be affected by recall bias. However, these studies did not mention any funding or publication bias. Comparing studies (Alosco *et al.*, 2014 and Bednarska-Makaruk *et al.*, 2019), we can see that the Makaruk study, despite its small sample size, included patients with different types of dementia^12,19^. We did not find much data on how adiponectin and leptin levels affect the different types of dementia, rather most information was combined under the umbrella term ‘cognitive decline’. We also focused mainly on the overall cognitive decline. However, the review conducted by Dye *et al.* ^11^ differentiated between the long-term and short-term effects of cognitive decline. This is a unique strength of our study as most studies published so far did not discuss the long-term and short-term effects in detail. The RCT conducted by Espeland *et al*.^10^, with its large sample size of 1091 subjects, has shown that obese individuals manifest higher cognitive decline than non-obese ones. Hence, comparing this study with those that have a small sample size has also well established that obesity and cognitive decline are linked^19,24^. Kiliaan *et al*.’s^15^ work further supports this conclusion.

In this paper, we concentrated mainly on the action of adiponectin and leptin in linking obesity with cognitive decline. Whereas most studies to this day focus on three different points of view. Firstly, how obesity is linked to dementia, secondly, how adiponectin and leptin dysregulation is developing cognitive decline. And thirdly, how different diseases like MetS, T2 DM and insulin resistance, obesity, heart failure, etc. develop dementia by several adipokines, inflammatory markers, or other hormones^11,12,19,23^. We did not diverge into discussing other pathologies adding to the uniqueness of our study. However, despite its small size, Bednarska-Makaruk *et al.*’s^19^ study focused on not just leptin and adiponectin but resistin, pro-inflammatory markers, and vitamins in connecting obesity with cognitive decline. In discussing adiponectin and leptin, we again focused on the molecular mechanisms carefully explaining the highly complex relationships between obesity and dementia. Hence, this paper is an excellent resource for learning and teaching pathology on a molecular level and can be considered useful while formulating novel research questions and refined hypotheses. For future studies, it would be interesting to look further into which has a bigger effect (adiponectin, leptin, inflammatory markers, or other hormones) in its role of associating dementia with obesity. The main graphic characteristics, such as age, gender, race, ethnicity, etc., require stratification in future studies too to be able to make more comprehensive conclusions on the significance of adiponectin and leptin in linking these two conditions. Another study can be done to focus solely on dementia rather than the ‘umbrella term’ cognitive decline. In addition, the focus is needed on the improvements of preventive modalities as many of them have significant side effects, making them difficult to use for people. Thus, comparing this study with the rest, it can be said that this review has added crucial information to the investigation on this matter.

## Conclusion

In this systematic review article, we have demonstrated that obesity is correlated with an increased likelihood of cognitive decline. Besides, we have also discussed the protein hormones adiponectin and leptin, which are the major associated factors between obesity and cognitive dysfunction. Adiponectin prevents obesity through energy expenditure and fatty acid catabolism. It further promotes neurogenesis and synaptic function. Moreover, leptin prevents obesity by promoting satiety and reducing hunger and insulin sensitivity. Whereas, it protects memory and cognitive function by preventing neurodegeneration and obesity-induced leptin resistance. With the disruption of these modulations, harmful symptoms begin. Therefore, it can be concluded that both adiponectin and leptin have a safeguarding role against obesity and cognitive frailty. All in all, the therapeutic intercession of adiponectin and leptin, lifestyle modifications such as exercise, weight reduction, and diet alteration together with cognitive restorative activities shield against cognitive dysfunction. Hence, further studies should be carried out in the mentioned areas.

## Ethical approval

Not applicable.

## Consent

Not applicable.

## Sources of funding

This review received no specific grant from any funding agency in the public, commercial, or not-for-profit sectors.

## Author contribution

N.T. and N.K. conceptualized the method of the study and supervised article inclusion–exclusion, data extraction, analysis, synthesis, and manuscript writing. All authors contributed to various aspects of article inclusion, data extraction, analysis, synthesis, and manuscript writing. N.T. and N.K worked on the final revision under the supervision of N.T.

## Conflicts of interest disclosure

The authors declare that there are no conflicts of interest.

## Guarantor

Nishat Tasnim, first and the corresponding author.

## Provenance and peer review

Not commissioned, externally peer-reviewed.
